# Therapeutic Benefits of Induced Pluripotent Stem Cells in Monocrotaline-Induced Pulmonary Arterial Hypertension

**DOI:** 10.1371/journal.pone.0142476

**Published:** 2016-02-03

**Authors:** Wei-Chun Huang, Meng-Wei Ke, Chin-Chang Cheng, Shih-Hwa Chiou, Shue-Ren Wann, Chih-Wen Shu, Kuan-Rau Chiou, Ching-Jiunn Tseng, Hung-Wei Pan, Guang-Yuan Mar, Chun-Peng Liu

**Affiliations:** 1 Cardiovascular Medical Center, Kaohsiung Veterans General Hospital, Kaohsiung, Taiwan; 2 School of Medicine, National Yang-Ming University, Taipei, Taiwan; 3 Department of Physical Therapy, Fooyin University, Kaohsiung, Taiwan; 4 Department and Institute of Pharmacology, National Yang-Ming University, Taipei, Taiwan; 5 Department of Education and Research, Taipei Veterans General Hospital, Taipei, Taiwan; 6 Department of Medical Education and Research, Kaohsiung Veterans General Hospital, Kaohsiung, Taiwan; 7 Department of Emergency, Kaohsiung Veterans General Hospital, Kaohsiung, Taiwan, People's Republic of China; University of Illinois College of Medicine, UNITED STATES

## Abstract

Pulmonary arterial hypertension (PAH) is characterized by progressive increases in vascular resistance and the remodeling of pulmonary arteries. The accumulation of inflammatory cells in the lung and elevated levels of inflammatory cytokines in the bloodstream suggest that inflammation may play a role in PAH. In this study, the benefits of induced pluripotent stem cells (iPSCs) and iPSC-conditioned medium (iPSC CM) were explored in monocrotaline (MCT)-induced PAH rats. We demonstrated that both iPSCs and iPSC CM significantly reduced the right ventricular systolic pressure and ameliorated the hypertrophy of the right ventricle in MCT-induced PAH rats in models of both disease prevention and disease reversal. In the prevention of MCT-induced PAH, iPSC-based therapy led to the decreased accumulation of inflammatory cells and down-regulated the expression of the *IL-1β*, *IL-6*, *IL-12α*, *IL-12β*, *IL-23* and *IFNγ* genes in lung specimens, which implied that iPSC-based therapy may be involved in the regulation of inflammation. NF-κB signaling is essential to the inflammatory cascade, which is activated via the phosphorylation of the NF-κB molecule. Using the chemical inhibitor specifically blocked the phosphorylation of NF-κB, and *in vitro* assays of cultured human M1 macrophages implied that the anti-inflammation effect of iPSC-based therapy may contribute to the disturbance of NF-κB activation. Here, we showed that iPSC-based therapy could restore the hemodynamic function of right ventricle with benefits for preventing the ongoing inflammation in the lungs of MCT-induced PAH rats by regulating NF-κB phosphorylation.

## Introduction

Pulmonary arterial hypertension is defined as a progressive increase in pulmonary vascular resistance leading to right ventricular failure and premature death [1]. Endothelial injury is associated with PAH, whereby aberrantly secreted vasodilators or vasoconstrictors and the production of cytokines or growth factors result in the recruitment of monocytes that differentiate into macrophages within impaired lesion sites [2–4]. Intense perivascular inflammation accompanied by arterial medial hypertrophy and pulmonary vascular remodeling have been observed in the lungs of MCT-induced PAH rats [5–7]. Elevated circulating cytokine and chemokine levels have been reported in PAH. Relatedly, the treatment of underlying inflammation has been shown to alleviate PAH-related symptoms. Taken together, these results suggest a role for inflammation in the pathogenesis of PAH [8–10].

Macrophages, which are generated from circulating monocytes, further differentiate into either classically activated M1 macrophages or alternatively activated M2 macrophages depending on the milieu of inflammation [11]. *In vitro* polarized M1 macrophages produce inflammatory cytokines (IL-1β, IL-6, IL-12/23, and TNF-α) and act as effector cells that participate in polarized Th1 immune responses, thereby destroying extracellular matrix, stimulating apoptosis, and promoting cellular immunity against intracellular parasites and tumors [11–13].

There is no cure for PAH. Advanced medical therapies substantially improve the survival rate and quality of life of patients with PAH; however, many unsuccessful cases persist [14–18]. Pulmonary vascular remodeling and microvascular loss are viewed as hallmarks of PAH; regenerative cell therapy has thus been proposed as a novel treatment. In MCT-induced PAH models, endothelial progenitor cells (EPCs) prevented the increase of right ventricular systolic pressure, whereas animals treated with EPCs transduced with human endothelial nitric oxide synthase (eNOS) exhibited a significant reversal of PAH [19]. Furthermore, the administration of early-EPCs has been shown to effectively prevent PAH onset in athymic rats via an immune system-dependent mechanism that potentially involves the stimulation of NK cells [20]. The sublingual vein injection of bone marrow-derived mesenchymal stem cells (BM-MSCs) into a rat model over the course of 2 weeks significantly improved the lung and heart injuries caused by left-to-right shunt-induced PAH, resulted in enhanced angiogenesis and decreased the levels of pulmonary vascular remodeling and inflammation [21]. Additional stem cell types have been proposed for the treatment of both myocardial infarction and PAH, including intrinsic cardiac stem cells [22–24], embryonic stem cells (ESCs) [25–27], and pediatric hematopoietic stem cells [28].

Induced pluripotent stem cells (iPSCs) are reprogrammed from adult somatic cells via the transduction of defined transcription factors; evidence suggests that iPSCs are virtually indistinguishable from ESCs [29, 30]. Studies have demonstrated the ameliorative effects of iPSCs via a paracrine mechanism with respect to both cardiac function after myocardial infarction [31] and retinal oxidative damage [32]. Interestingly, studies performed by Chiou et al. suggested that iPSCs attenuate the severity of endotoxin-induced acute lung injury or ventilator-induced lung injury via the suppression of NF-κB (nuclear factor kappa-light-chain-enhancer of activated B cells) signaling and neutrophil accumulation [33, 34].

In this study, iPSCs were used in a novel therapy for the treatment of MCT-induced PAH rats. We are the first to explore whether iPSC-based therapy benefits the hemodynamic function of the right ventricle and underlying inflammation in MCT-induced PAH.

## Materials and Methods

### Ethics Statement

The manipulation of animals throughout this study was approved by the Institutional Animal Care and Use Committee, Kaohsiung Veterans General Hospital, Kaohsiung, Taiwan; the principles of the Animal Protection Act (Council of Agriculture, Executive Yuan) were followed. The IACUC Approval No. is vghks-2013-A010.

The Human Research Committee of Kaohsiung Veterans General Hospital approved the study protocol. And, the written informed consents from each of the individuals were obtained.

### Culture of murine induced pluripotent stem cells

Murine iPSCs were kindly provided by Dr. Shih-Hwa Chiou [32–34]. Mouse embryonic fibroblasts (MEFs), used as feeder cells, were cultured to confluence and subsequently treated with 10 μg/mL mitomycin C for 3 h to inhibit proliferation. The cells were rinsed with 1X phosphate buffer. The iPSCs were seeded onto the feeder layer in DMEM medium [20% fetal bovine serum (FBS), 100 U/mL penicillin, 100 μg/mL streptomycin, 2 mM GlutaMAX, 1X non-essential amino acid (NEAA), 12 mM 4-(2-hydroxyethyl)-1-piperazineethanesulfonic acid (HEPES), 1.2 mM sodium pyruvate and 0.0004% β-mercaptoethanol] and incubated at 100% humidity and 5% CO_2_. The culture medium was replaced daily. To obtain iPSC-conditioned medium (iPSC CM) for the treatment of MCT-induced PAH rats, the culture medium was replaced with serum-free basal medium containing high glucose DMEM supplemented with 10 mM NEAA and 0.3% leukemia inhibitory factor (LIF); the cells were cultured for 48 h [34].

### Alkaline phosphatase activity

The alkaline phosphatase activity of iPSCs was detected with an Alkaline Phosphatase kit (Sigma-Aldrich, St Louis, MO USA). As per the manufacturer’s instructions, iPSCs grown on cover glass were fixed with a citrate-acetone-formaldehyde solution for 30 seconds at room temperature. The cells were washed with 1X PBS and incubated in FRV-Alkaline Solution for 15 minutes at room temperature. The iPSCs were counterstained with hematoxylin for 2 minutes. The cover slide was rinsed with tap water and mounted in aqueous mounting media.

### Experimental design

Male rats of the SD strain weighing 200–250 grams were purchased from BioLASCO Taiwan Co., Ltd (Taipei, Taiwan R.O.C.). The animal PAH model was achieved via the subcutaneous injection of MCT (60 mg/kg), whereas the control animals received only PBS (1 mL). For the iPSC-based therapy, MCT-induced PAH rats were treated with either iPSCs or iPSC CM. The rats receiving transplanted iPSCs were anesthetized via an intramuscular injection of Zoletil^™^ (40 mg/kg) via the tail vein once per week with 2 x 10^6^ iPSCs suspended in PBS (1 mL). The rats treated with iPSC CM received daily intraperitoneal injections of iPSC CM (1 mL) until the end of the study.

The iPSC-based therapy could be further divided into either prevention or reversal protocols. For prevention, the rats were given iPSC-based therapy on the same day as the MCT injection, following either transplanted iPSCs once per week or iPSC CM every day until the end of the study. For reversal, the rats began to receive iPSCs or iPSC CM after 14 days of MCT, and the administration of iPSCs or iPSC CM was the same as above. MCT-induced PAH rats were randomly assigned into prevention or reversal treatment.

### Hemodynamic measurement

The right ventricular systolic pressure (RVSP) of rats was measured using a PowerLab/8_SP_ instrument (ADInstruments Ltd. Dunedin, New Zealand). In brief, the rats were anesthetized with Zoletil^™^ and the chest area was shaved and sterilized with 75% ethanol. An endotracheal intubation was performed to maintain the cardiopulmonary function of the anesthetized rat during the open-chest surgery and the RVSP recording. The right ventricle was exposed and catheterized with a polyethylene tube filled with heparinized PBS, which was connected to the PowerLab/8_SP_ instrument. The RVSP was recorded for at least 6 seconds with a stable blood pressure waveform for each rat.

### Immunocytochemistry

The cells grown on the cover slides were rinsed with 1X PBS and fixed with 4% formaldehyde (in 1X PBS) for 15 minutes. The cells were subsequently washed 3x (5 minutes each) with 1X PBS. The cells were permeabilized with 0.05% Triton X-100 (in 1X PBS) solution for 10 minutes and washed 3x with 1X PBS (5 minutes each). The cells were blocked with 10% normal goat serum (in 1X PBS) for 1 h. Primary antibody was diluted in blocking media, and the cells were incubated overnight at 4°C. Information regarding the antibodies used is described in S1 Table. The cells were washed 3x (5 minutes each) with 1X PBS and incubated in fluorochrome-conjugated secondary antibody for 2 h at room temperature. The nuclei were counterstained with 4',6-diamidino-2-phenylindole (DAPI) for 10 minutes. The cover slides were immersed in anti-fade mounting media and imaged using a laser confocal microscope.

### Western blotting

Protein samples (20 μg each) were loaded onto an SDS-polyacrylamide gel and blotted onto a PVDF membrane. The membranes were blocked in BlockPRO^™^ Blocking buffer (Visual Protein Biotechnology Corp., Taiwan) at room temperature for 30 minutes. Primary and secondary antibodies were diluted in blocking buffer according to the manufacturers’ instructions. Information regarding the antibodies used is described in S1 Table. The target proteins were incubated overnight in primary antibody at 4°C. The membranes were washed briefly in 1X PBS and incubated in HRP-conjugated secondary antibody at room temperature for 2 h. For signal detection, the membranes were washed 3x in 1X PBS (5 minutes each) and subsequently incubated with Clarity^™^ Western ECL Substrate (Bio-Rad Laboratories, Inc., CA USA). The images were developed on FUJIFILM Super RX medical film (Fujifilm Holdings Corp., Japan).

### Enzyme—linked immunosorbent assay (ELISA)

The IL-1β (or TNFα) levels were measured using the Human IL-1 beta (or TNF alpha) ELISA Ready-SET-Go^®^ kit (eBioscience, INC., San Diego, CA USA) according to the manufacturer’s instructions. The capture antibody was pre-coated in designated wells of a 96-well plate at 4°C overnight. The wells were washed 5x (1 minute each) with 0.05% Tween 20 in PBS and blocked with 1X Assay Diluent at room temperature for 1 h. Sample supernatant (100 μL) was added, and the wells were incubated at room temperature for 2 h. The wells were subsequently washed and incubated with detecting antibody for 1 h. The signals were developed with Avidin-HRP, and the reaction was stopped with 2N H_2_SO_4_ solution (50 μL). Signal intensity was detected using a Fluoroskan Ascent^™^ FL Microplate Fluorometer (Thermo Scientific^™^, MA USA).

### Immunohistochemistry

Lung tissue sections of 5 μm thickness were deparaffinized via xylene soak (2x 7 minutes). The samples were rehydrated in serial ethanol solutions (100% to 50%, 2 minutes each). For antigen retrieval, the samples were immersed in pre-heated boiling citrate buffer (10 mM citric acid, 0.05% Tween 20, pH 6.0) for 20 minutes. The samples were cooled, rinsed in 1X PBS, blocked with 10% normal goat serum for 30 minutes, and incubated in primary antibody diluted in blocking buffer at 4°C overnight. Information regarding the antibodies used is described in S1 Table. The samples were washed briefly in 1X PBS and incubated in horseradish peroxidase (HRP)-conjugated secondary antibody for 2 h. Signals were detected via 3,3'-diaminobenzidine (DAB) solution. The nuclei were counterstained with hematoxylin solution for 5 minutes. Sections were washed with 1X PBS and mounted in Aquatex^®^ for imaging.

### Semi-quantitative real time—polymerase chain reaction (RT-PCR)

Complementary DNA (cDNA) templates were generated from 1 μg total RNA extract using SuperScript^®^ VILO^™^ (Invitrogen^™^, CA USA) according to the manufacturer’s instructions. Gene-specific primer pairs are listed in S2 Table. cDNA templates were titrated to 50 ng/μL, and 1 μL was mixed with the components provided by Fast SYBR^®^ Green Master Mix (Applied Biosystems, CA USA). The StepOnePlus^™^ System was used with the associated v2.2.2 software (Applied Biosystems^®^ by life technologies, NY USA).

### Human macrophages

Human macrophages were derived from peripheral blood mononuclear cells (PBMCs) [12, 35, 36]. Blood was donated from healthy male adults, and PBMCs were isolated using Ficoll-Paque PLUS (GE Healthcare, Uppsala, Sweden) and cultured in RPMI 1640 medium [20% FBS, 1% P/S and macrophage colony stimulating factor (M-CSF)] for 7 days of macrophage differentiation. For the purpose of culturing pro-inflammatory polarized M1 macrophages, the culture medium was replaced with RPMI 1640 supplemented with 5% FBS, 100 ng/mL lipopolysaccharide (LPS) and 20 ng/mL interferon γ (IFNγ) for an additional 24 h. All protocols were approved by the Institutional Review Board of Kaohsiung Veterans General Hospital, Kaohsiung, Taiwan.

### Statistical analysis

Each independent experiment consisted of at least 3 animals studied simultaneously. The data are expressed as the means ± SD. One-way ANOVA and Scheffé Post Hoc tests were used in the context of multiple comparisons. Significance was defined as *p<*0.05. IBM^®^ SPSS^®^ Statistics Version 21 software was used for all statistical analyses.

## Results

### Characterization of induced pluripotent stem cells (iPSCs)

**Fig 1:**
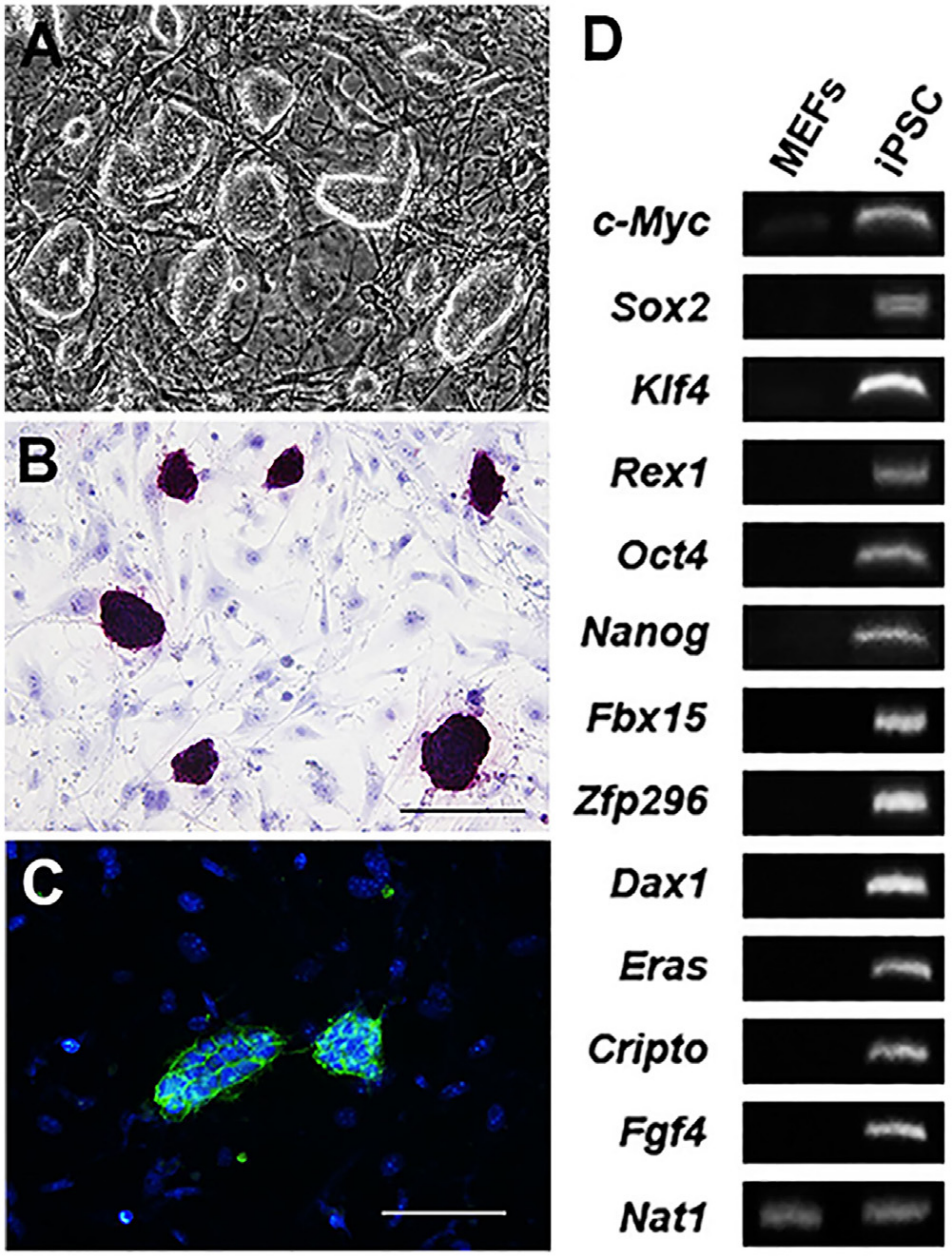
Characterization of murine induced pluripotent stem cells. Murine iPSCs were plated onto mitomycin c-treated MEFs for 3 days and formed cobblestone-like colonies. (B) iPSCs demonstrated specific and strong alkaline phosphatase activity. Scale bar = 100 μm. (C) Immunocytochemistry revealed the expression of iPSC marker SSEA-1. Scale bar = 100 μm. (D) Gel electrophoresis demonstrates the semi-quantitative RT-PCR results for several key genes unique to iPSCs. *Nat1* served as an internal control.

Murine iPSCs were grown on MEFs *in vitro* for 3 days. The colonies exhibited a cobblestone-like morphology typical of iPSCs and stained positively for alkaline phosphatase activity. Immunocytochemistry revealed the positive expression of pluripotent stem cell marker SSEA-1 (Fig 1A to 1C). Semi-quantitative RT-PCR demonstrated gene expression typical of iPSCs (Fig 1D).

### iPSC-based therapy ameliorated hemodynamic alterations in MCT-induced PAH

**Fig 2:**
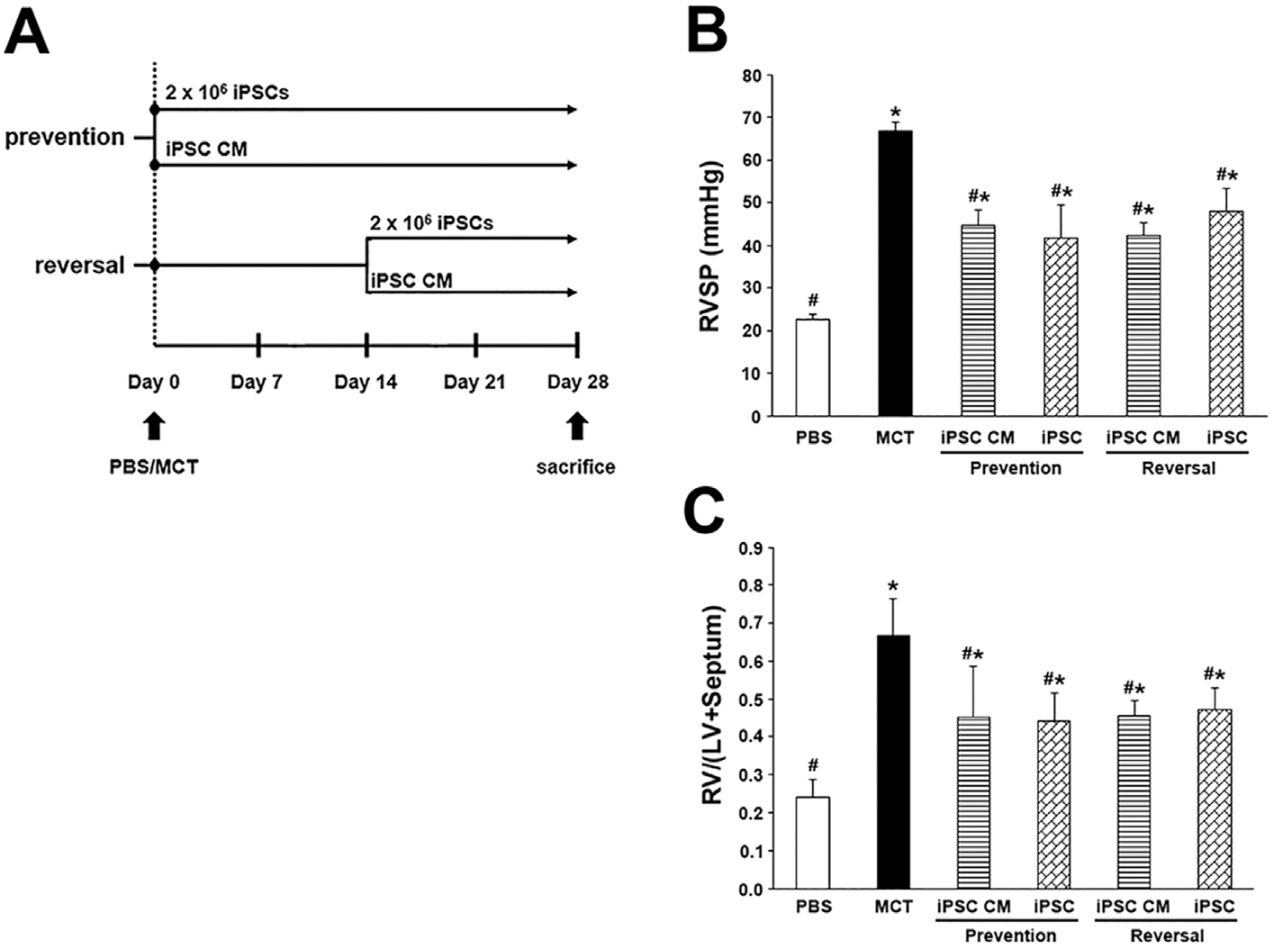
Effect of iPSC-based therapy on RVSP and the weight ratio of RV/(LV+Septum). Representative scheme of iPSC-based therapy on MCT-induced PAH. (B) Hemodynamic changes in RVSP were analyzed across animal groups at day 28 prior to sacrifice. (C) RV hypertrophy was measured by comparing the weight ratio of RV/(LV+Septum) of different groups. The results are shown as the mean ± SD for 6 rats of each group. *p<0.05 vs. PBS group; ^#^*p<*0.05 vs. MCT group.

As shown in Fig 2B and 2C, the administration of MCT resulted in significant pathologic values for RVSP (66.81 ± 2.02 mmHg) and severe hypertrophy of the right ventricle (RV) in rats. The MCT-induced PAH rats that received iPSC-based therapy for prevention or reversal both exhibited significant improvements regarding RVSP and RV hypertrophy. There were no significant differences regarding hemodynamic value or RV hypertrophy improvement between the prevention group and the reversal group; the following experiments focused on the prevention protocol of the iPSC-based therapy in MCT-induced PAH.

### Histological examination of the effects of iPSC-based therapy in the pulmonary arteries of MCT-induced PAH rats

The morphology of the pulmonary arteries was examined via EVG stain and further evaluated by measuring the extent of hypertrophy of the media layer.

**Fig 3:**
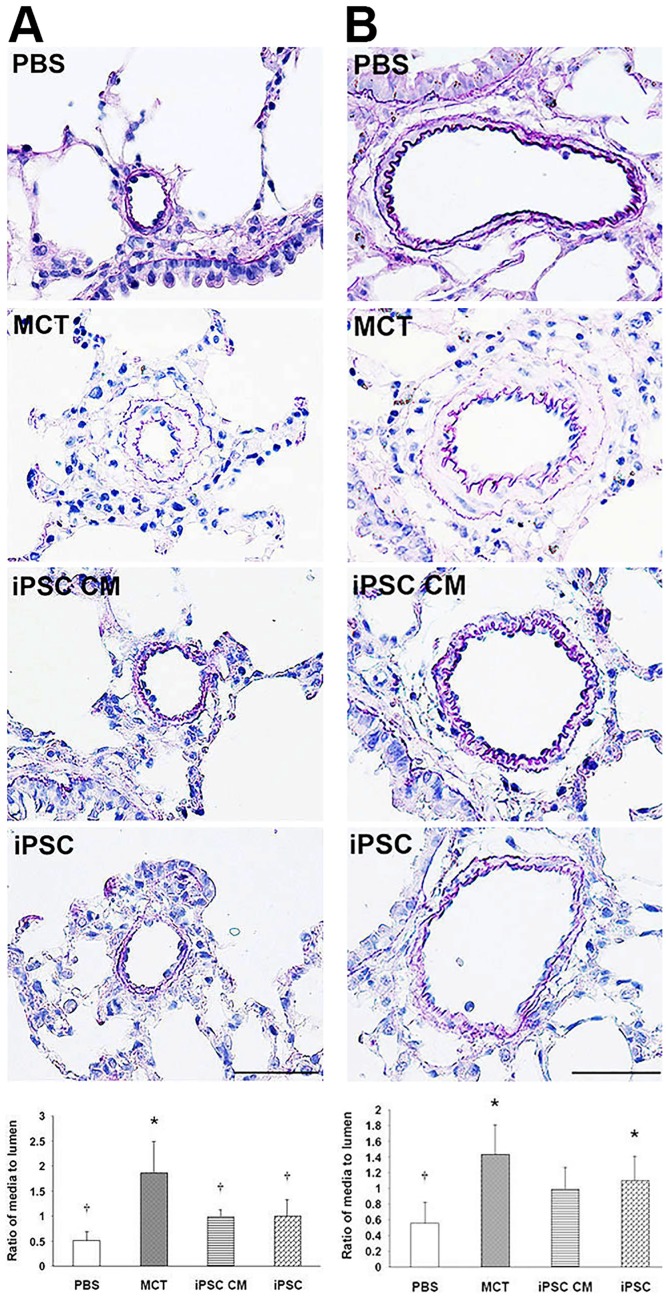
EVG analysis of pulmonary artery hypertrophy. The progressive narrowing and thickening of blood vessels (particularly beginning in arterioles) is typical in PAH. We performed EVG staining on paraffin-embedded lung sections. Blood vessels were divided into two classes according to their diameter: (A) < 100 μm and (B) > 100 μm. The thickness of blood vessels was evaluated by calculating the ratio of the area of the media layer to that of the lumen. Our findings were most dramatic in blood vessels under 100 μm in diameter; iPSCs treatments significantly prevented the progressive thickening of the arterioles. The area ratio of the media to the lumen was represented as the means ± SD, and the value for each group was averaged over 10 vascular images taken from different fields. Scale bar = 100 μm. *p<0.05 vs. PBS group; ^†^*p<*0.05 vs. MCT group.

Taking into consideration areas with a vascular diameter of under 100 μm, the ratios of the area of the media layer to those of the lumen in the treatment groups for PBS, MCT, iPSC CM and iPSCs were 0.51 ± 0.17, 1.88 ± 0.61, 1.02 ± 0.31 and 1.10 ± 0.61, respectively. In areas with vascular diameters greater than 100 μm, the area ratios of the media to the lumen were 0.56 ± 0.27 (PBS), 1.44 ± 0.37 (MCT), 1.00 ± 0.27 (iPSC CM) and 1.03 ± 0.43 (iPSCs). Importantly, both of the groups treated with iPSC CM and iPSCs demonstrated a significant improvement (*p<*0.05) in media layer hypertrophy (Fig 3A and 3B).

### iPSC therapy-mediated attenuation of inflammation in the lung tissue of MCT-induced PAH rats

The immunohistochemical analysis of paraffin-embedded murine lung tissue was performed for two inflammatory markers on the cell surface (CD68 and MHC-II).

**Fig 4:**
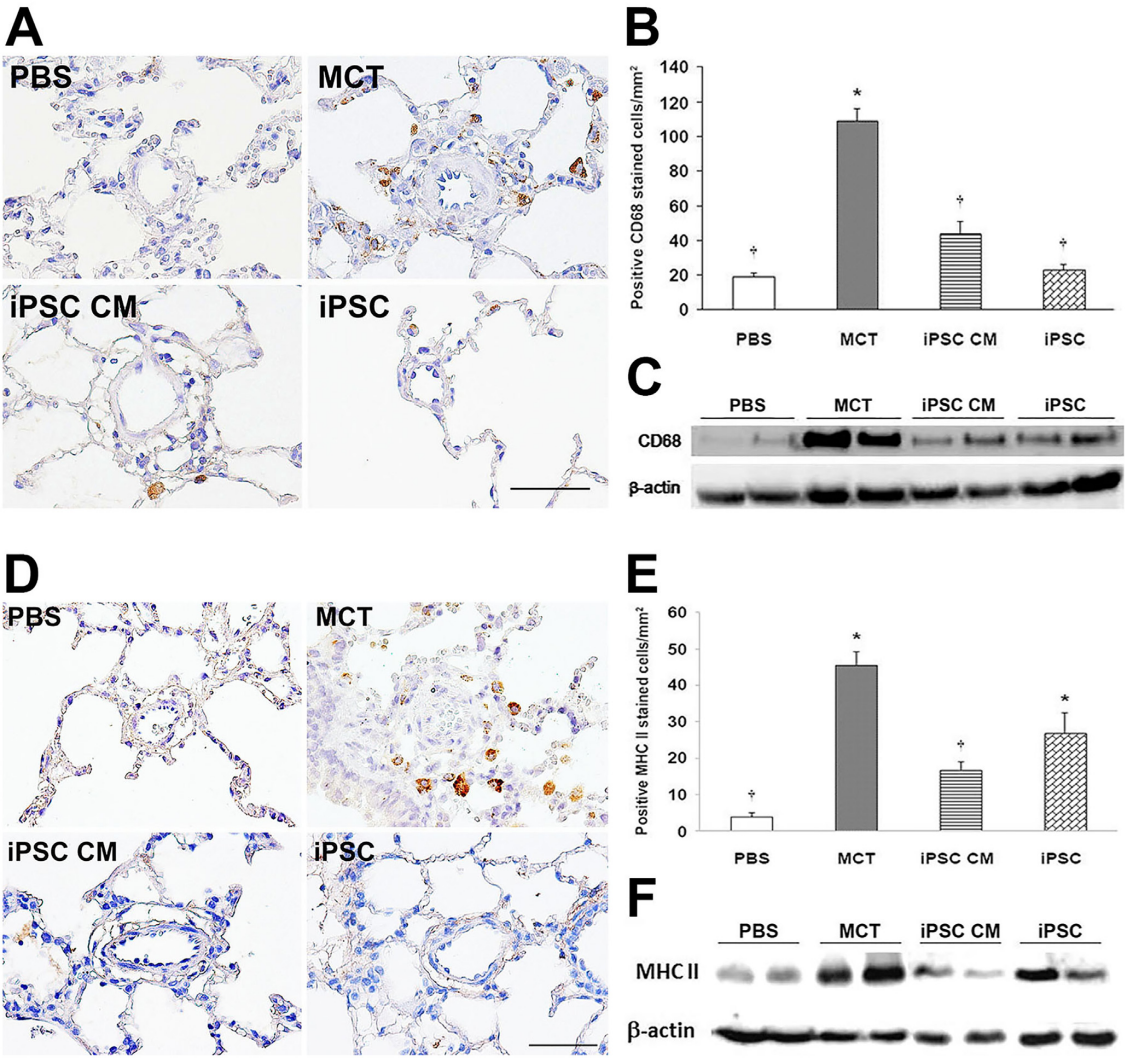
iPSC-based therapy attenuated inflammation in the lung tissue of MCT-induced PAH rats. Inflammation in the lungs of MCT-induced PAH rats was analyzed via immunohistochemistry of inflammatory markers in paraffin-embedded sections. (A) Positive staining for macrophage marker CD68. (B) Quantification of CD68 stain calculated as the number of positive cells per sq. millimeter from 10 separate images of different fields; data are represented as the means ± SD. (D-E) The same protocol was used to detect MHC-II; the results were confirmed via western blotting (C and F, CD68 and MHC-II, respectively). iPSC-based therapy attenuated the level of inflammation in MCT-induced PAH rats. Scale bar = 100 μm. *p<0.05 vs. PBS group; ^†^*p<*0.05 vs. MCT group.

CD68, which is ubiquitously expressed in inflammatory tissues, stained positively throughout the sections of the MCT-treated group (108.8 ± 6.9 positive cells/mm^2^) compared with the PBS group (18.7 ± 2.4), iPSC CM group (44.1 ± 6.6) and iPSCs group (22.9 ± 3.1) (Fig 4A and 4B). MHC-II staining was present in the PBS (3.9 ± 1.1 positive cells/mm^2^), MCT (45.5 ± 3.7), iPSC CM (16.8 ± 2.3) and iPSCs (27.0 ± 5.4) groups, respectively (Fig 4D and 4E). These results were confirmed via western blotting (Fig 4C and 4F). This finding strongly suggests that treatment with either iPSC CM or iPSCs significantly ameliorated inflammation in the lungs of MCT-induced PAH rats.

### Downregulation of the expression of inflammatory-associated genes mediated by iPSC-based therapy

**Fig 5:**
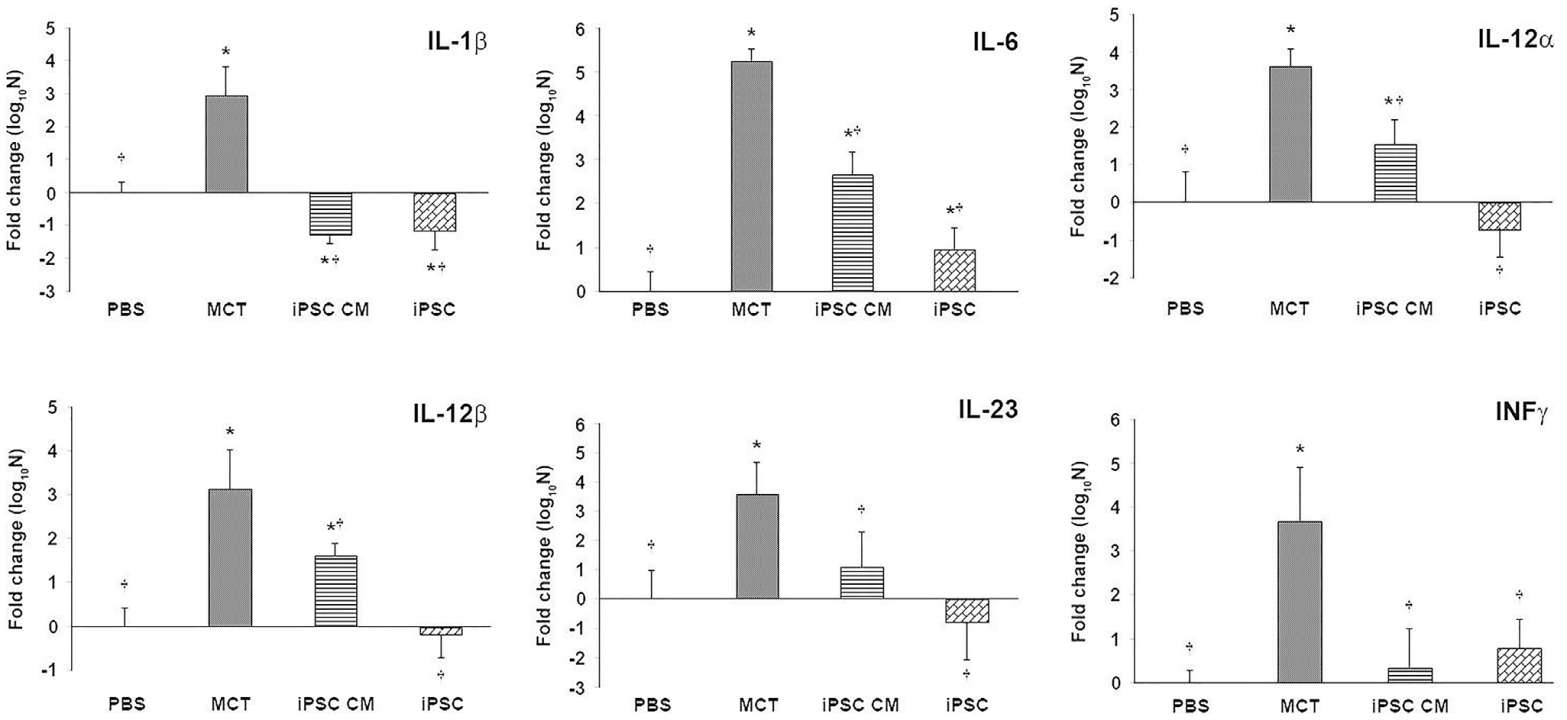
iPSC-based therapy downregulated the expression of proinflammatory genes in the lungs of MCT-induced PAH rats. Semi-quantitative RT-PCR (qRT-PCR) was used to detect the expression of several proinflammatory genes including *IL-1β*, *IL-6*, *IL-12α*, *IL-12β*, *IL-23* and *INFγ*. RNA extracted from the lung tissue of the MCT-induced PAH rats and controls was reverse transcribed for semi-quantitative RT-PCR. The data were normalized according to *36b4* mRNA levels and represented as a value relative to the PBS control group. The results are shown as the means ± SD for 5–8 rats per group. *p<0.05 vs. PBS group; ^†^*p<*0.05 vs. MCT group.

Lung tissue inflammation was analyzed using the expression of several pro-inflammatory genes. Semi-quantitative RT-PCR data revealed the significantly reduced expression of key pro-inflammatory genes (*IL-1β*, *IL-6*, *IL-12α*, *IL-12β*, *IL-23* and *IFNγ*) in both the iPSC CM and iPSCs groups (Fig 5).

### The transplantation of iPSCs prevented the secretion of cytokines from human macrophages *in vitro*

**Fig 6:**
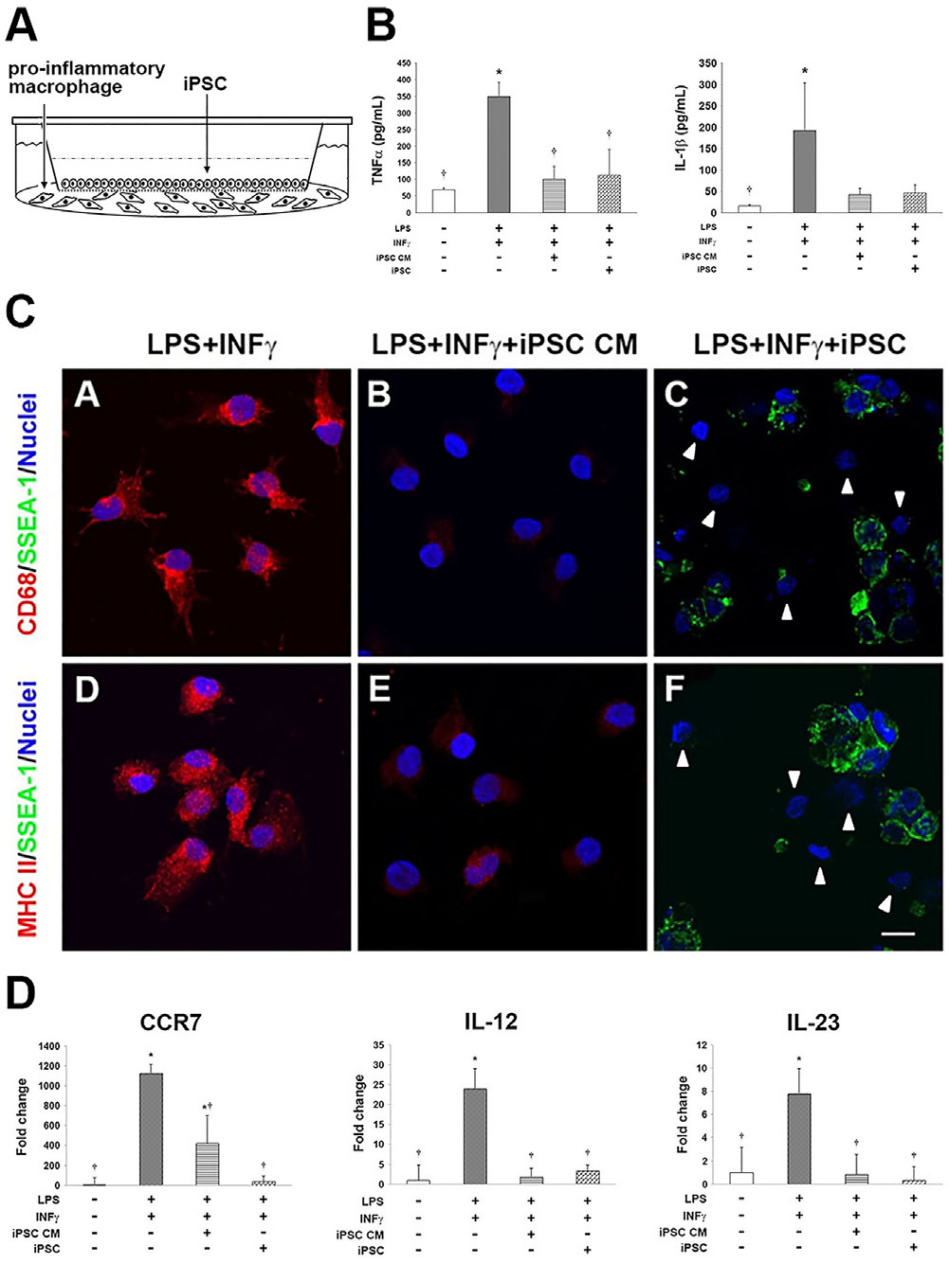
iPSC-based treatment suppressed inflammation in polarized classical human macrophages. The effects of murine iPSCs on inflammation were evaluated in the context of co-culture with polarized classical human macrophages. Primitive human macrophages were differentiated from PBMCs and subsequently polarized (via the stimulation of LPS and IFNγ) to become proinflammatory M1 macrophages. To examine paracrine effects of iPSCs *in vitro*, cells were separated using a permeable membrane (A), thereby mimicking the conditioned media treatment in rats. (B) Secretion of the inflammatory cytokines TNFα and IL-1β was measured via ELISA. Data were accumulated from 3–5 independent assays. (C) The immunocytochemical analysis of inflammatory markers CD68 and MHC-II was performed on co-cultured iPSCs and M1 macrophages. CD68 and MHC-II (Texas Red) and iPSC-specific marker SSEA-1 (FITC) are shown. Nuclei were counterstained with Hoechst 33342. (D) Genes specifically expressed in M1 macrophages were compared across groups of iPSC-based treatments using semi-quantitative RT-PCR. The results are represented as the means ± SD; values were normalized according to *36b4* mRNA levels and represented as the fold change relative to the PBS control group. *p<0.05 vs. PBS group; ^†^*p<*0.05 vs. MCT group. Scale bar = 10 μm.

Induced pluripotent stem cells were either directly co-cultured with human pro-inflammatory M1 macrophages (akin to iPSCs animal group) or separated using a 0.4 μm Transwell^®^ permeable membrane (akin to the iPSC CM animal group, Fig 6A). Inflammation was assessed in terms of tumor necrosis factor α (TNFα) and IL-1β levels by using ELISA. In both the co-cultured and membrane groups, the levels of secretory TNFα were significantly lower than those in the positive group. Similar changes were observed for the IL-1β concentration (Fig 6B).

Furthermore, an immunocytochemical analysis demonstrated the faint staining of CD68 and MHC-II in both the co-cultured and membrane groups (Fig 6C). A semi-quantitative RT-PCR analysis supported these results, revealing a downregulation in the expression levels of *CCR7*, *IL-12*, or *IL-23* in both iPSC treatment groups (Fig 6D).

### iPSC-based therapy ameliorated PAH-associated inflammation via the inhibition of NF-κB phosphorylation

NF-κB is a crucial transcription factor with respect to the immune response. Its phosphorylation and nuclear translocation drives the expression of several inflammatory cytokine genes. Thus, we investigated whether iPSC-based therapy attenuated inflammation in the lungs of MCT-induced PAH rats by interfering with the phosphorylation of NF-κB.

**Fig 7:**
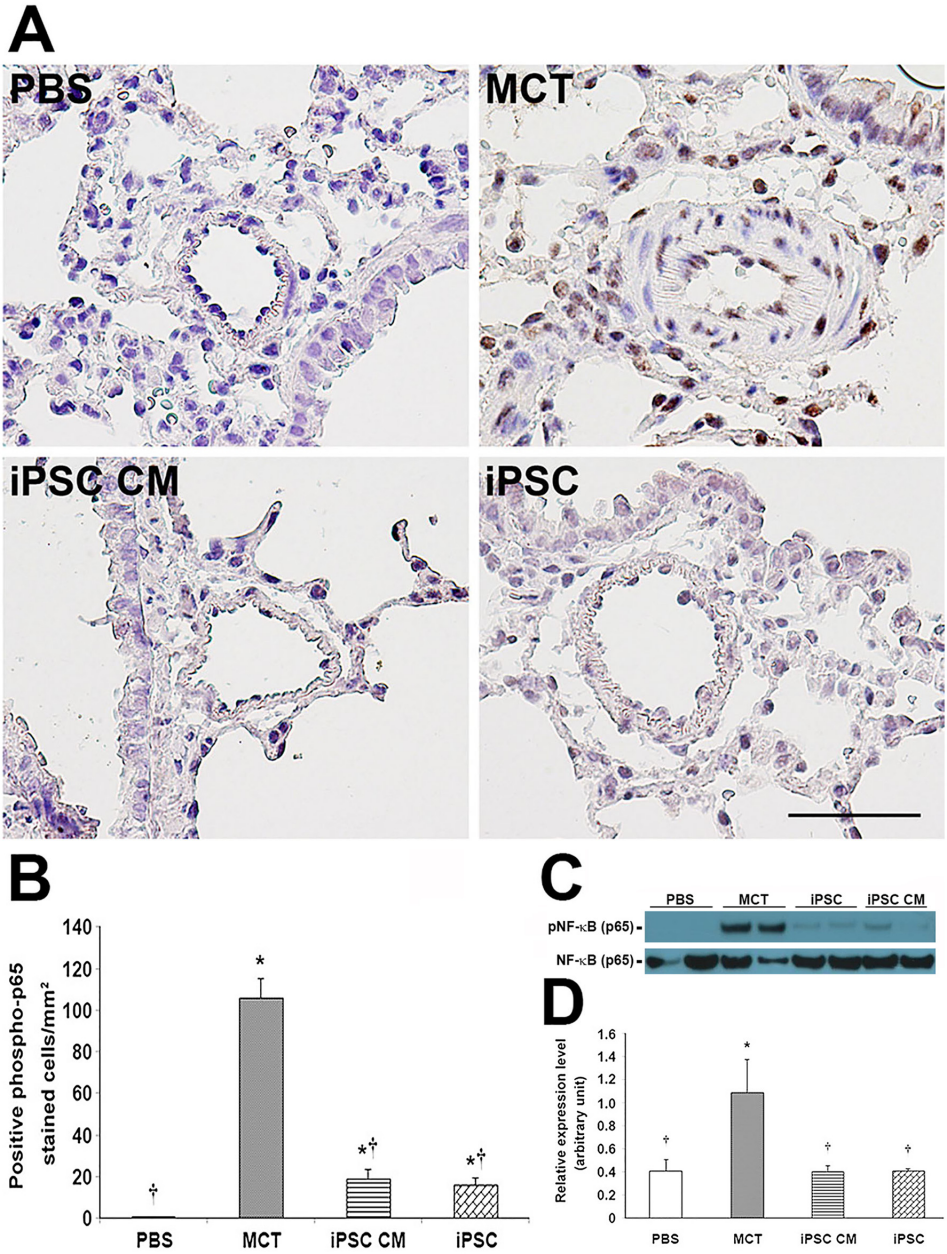
iPSC-based therapy regulated inflammation in MCT-induced PAH via the suppression of NF-κB phosphorylation. Phosphorylation of NF-κB protein was detected via immunohistochemistry with a monoclonal antibody specific to the phosphorylated form of the NF-κB p65 subunit. (C) The expression level of phosphorylated NF-κB protein relative to the total was confirmed via western blot. (B and D) Quantification of immunohistochemistry and western blot, respectively. Both iPSC treatments demonstrated significant inhibitory effects on NF-κB phosphorylation compared with MCT treatment. The phosphorylated NF-κB stain was quantified as the number of positive cells per sq. millimeter from 10 separate images of different fields; results are given as the means ± SD. *p<0.05 vs. PBS group; ^†^*p<*0.05 vs. MCT group. Scale bar = 100 μm.

Immunohistochemical analysis revealed a significant decrease in the number of cells expressing the phosphorylated p65 subunit of NF-κB in MCT-induced PAH rats receiving either iPSC CM or iPSCs (Fig 7A and 7B) compared with the MCT group. These results were confirmed via western blot (Fig 7C and 7D), thereby suggesting that iPSC-based therapy may attenuate inflammation in the lungs of MCT-induced PAH rats by disturbing NF-κB phosphorylation. *In vitro* human M1 macrophages co-cultured with iPSC-based treatments demonstrated a comparable effect of a specific inhibitor for NF-κB phosphorylation (S2 File).

## Discussion

In this study, we obtained promising results for iPSC-based therapy in MCT-induced PAH rats. IPSC-based therapy ameliorated hemodynamic changes and the corresponding hypertrophy of pulmonary arteries and RV. Furthermore, iPSC-based therapy reduced the expression of the inflammatory markers CD68, MHC-II, IL-1β, IL-6, IL-12α, IL-12β, IL-23 and IFNγ in a rat PAH model. We were also able to reproduce these findings in human polarized macrophages *in vitro*. These effects appear to be associated with the suppression of NF-κB phosphorylation.

The effect of iPSC-based therapy on PAH was first assessed by examining the hemodynamic values of the right ventricle. The progressive increase in pulmonary vascular pressure of PAH typically leads to a compensatory hypertrophy of the right ventricle in an attempt to overcome resistance. In our study, the calculated weight ratio of the RV/(LV+Septum) significantly decreased after iPSC-based therapy (Fig 2C); this was concurrent with a lower RV systolic pressure (Fig 2B). Also, there were no significant differences between the prevention and reversal groups receiving iPSC-based therapy in regard to improvements in RV hypertrophy (Fig 2C). A similar result was observed in the treatment of MCT-induced PAH rats with bone marrow-derived endothelial-like progenitor cells [18].

Additional studies suggested that PAH may arise from arteriolar occlusion via the apoptosis of endothelial cells [37–39]. Accordingly, EVG-stained pulmonary vascular specimens demonstrated reduced hypertrophy of the vascular wall after iPSC-based therapy, particularly in blood vessels with a diameter smaller than 100 μm (Fig 3). The efficacy of iPSC-based therapy with respect to PAH may be associated with maintaining the integrity of the vascular endothelium.

Furthermore, growing evidence suggested a role for inflammation in PAH in both animal models and clinical patients [7, 20, 40–43]. Stem cells were suggested as an alternative therapy for inflammatory disease. MSCs were demonstrated to possess anti-inflammatory effects via the suppression of interleukin-1β, MCP-1, and inducible nitric oxide synthase in a cerebral ischemic rat model [44, 45]. Similarly, iPSCs have been shown to be beneficial by attenuating inflammation in a murine model of acute asthma [46] and stroke-injured aged brain [47]. Anti-oxidative stress and the anti-inflammatory and anti-apoptotic properties of iPSCs have also been reported in an ischemia-reperfusion-induced acute kidney injury rat model [39]. In the present study, iPSC-based therapy downregulated the expression of several pro-inflammatory genes in an MCT-induced PAH rat model (Figs 4 and 5). Of note is CD68, which is an important macrophage marker [48]. Classically activated M1 macrophages display elevated expression levels of MHC-II, several inflammatory cytokines (IL-1β, TNF-α, IL-6, IL-12, and IL-23) and matrix metalloproteinases, thereby expressing an increased ability to induce inflammation [11, 12]. In the present study, treatment with either iPSCs or iPSC CM resulted in the decreased expression of CD68 or MHC-II in the lung tissues of MCT-induced PAH rats (Fig 4). Furthermore, the downregulated expression of pro-inflammatory genes identified via semi-quantitative RT-PCR validated the suppressive effect of iPSC-based therapy on inflammation in MCT-induced PAH (Fig 5). These results were further confirmed *in vitro* using iPSC-based therapy to treat polarized pro-inflammatory M1 macrophages (Fig 6A to 6D). We also provided direct evidence of injected iPSCs recruiting to the lung parenchyma around the thickened musculinized blood vessels of MCT-induced PAH rats (S1 Fig). However, the detailed mechanisms of the recruitment of injected iPSCs and the interaction with inflammatory cells require further investigation.

The NF-κB cascade was identified as an important signaling pathway for inflammation and is modulated by the classical activation of human macrophages [12]. Previous reports revealed NF-κB as a key factor in the signaling pathway mediating anti-inflammatory and anti-remodeling effects in the context of pulmonary hypertension [48, 49]. Though the exact molecular mechanisms have not been thoroughly described, the transcription factor NF-κB may underlie mechanisms of the iPSC-based treatment of endotoxin-induced acute lung injury in mice [34]. Furthermore, iPSC-based therapy was shown to decrease high tidal volume ventilation-induced inflammation, oxidative stress, and MIP-2 production via the inhibition of the NF-κB/NKRF pathways. Notably, the iPSC-conditioned medium revealed beneficial effects equal to those of iPSCs [32, 33]. In our study, the phosphorylated level of the NF-κB p65 subunit was lower in rats receiving iPSC-based therapy than rats treated with MCT alone (Fig 7).

To investigate why iPSC-based therapy attenuated inflammation in MCT-induced PAH, a unique band on SDS-PAGE belonging to the iPSC-conditioned medium was subjected to mass spectrometry. To our surprise, two major peptide sequences with significant values were identified (α1-antitrypsin (A1AT) and α2 HS-glycoprotein, S1 File). Both molecules were identified to have anti-inflammatory properties. A1AT is the most abundant member of the serpin superfamily. A1AT is in circulation and serves as primary acute-phase reactant in acute inflammation. Numerous studies have investigated the anti-inflammation mechanisms of A1AT, including its role in decreasing the expression of MHC-II and Toll-like receptor-2 and -4 in LPS-stimulated mouse pancreatic islet macrophages [50], inhibiting the production of pro-inflammatory cytokines in whole blood [51, 52], and reducing TNF-α secretion via the inhibition of TNF-α-converting enzyme activity to protect lung endothelial cells from inflammation injury [53]. Alpha2 HS-glycoprotein is also a common protein in the circulation that has been described as an inhibitor of calcification and a negative acute-phase reactant. Hepatic mRNA expression and the levels of α2 HS-glycoprotein in the serum were decreased during trauma and acute-phase systematic inflammation. Studies have also indicated that α2 HS-glycoprotein could suppress the activation of LPS-stimulated macrophages and could diminish the pro-inflammatory action of TNF-α by promoting the uptake of natural anti-inflammatory agents [54–56]. This may partially explain why iPSC-conditioned medium had comparable effects to those of iPSCs alone.

## Conclusions

Our findings demonstrate the potential for iPSC therapies (iPSCs or iPSC CM) with respect to PAH. We demonstrated an alleviation of the inflammation related to classically activated M1 macrophages in MCT-induced PAH in rats, a restoration of the integrity of pulmonary blood vessels and improved function of the right ventricle. Though more detailed pathogenic mechanisms of PAH are required, this research suggests that induced pluripotent stem cells may hold potential as a reliable therapy for patients suffering from PAH.

## Limitations

Regenerative cell therapy, such as that with endothelial progenitor cells, bone marrow-derived mesenchymal stem cells and embryonic stem cells, has been demonstrated to improve symptoms of pulmonary arterial hypertension. To date, this is a pioneer study using induced pluripotent stem cells as a source of regenerative cell therapy in PAH. Though the iPSC-based therapy provided valuable results in an MCT-induced PAH animal model, concerns regarding the use of iPS cells should be considered. (1) The study demonstrated significant effects of iPSC-based therapy in MCT-induced PAH rat models. However, this therapeutic effect may not necessarily translate to similar results in humans. (2) The protocols developed here were designed for the amelioration of MCT-induced PAH in rats via the transplantation of iPS cells or conditioned medium. However, early signatures of PAH are difficult to diagnose due to a lack of obvious clinical symptoms. Further applications of iPSC-based treatments of PAH should focus on whether the progression of ongoing PAH symptoms can be prevented or reversed. (3) The pathophysiology of PAH is complex, and a variety of cell types may contribute differently to PAH progression. The relationship between iPSCs and macrophages may not represent the entire situation with respect to an inflammatory reaction to iPSC-based therapy in PAH. This requires further investigation.

## Supporting Information

S1 Fig Injected iPS cells embedded in the parenchyma of lung tissue of MCT-induced PAH rat. Four hours prior to sacrifice, iPS cells were injected into the MCT-induced PAH rat via tail vein. Lung tissue was harvested for frozen sectioning and immunohistochemistry. Specimens from the MCT-induced PAH rats without the injection of iPS cells served as an antiserum control. Thickening blood vessels due to hyperplasia of smooth muscle cells were revealed via intensive stain of α-smooth actin (FITC, left panel). In specimens from MCT-induced PAH rats receiving iPS cells injection, the iPS cells were recognized by the antisera against SOX2 protein (Texas Red, right panel). Scale bar = 20 μm.
(TIF)

S1 File Secretome analysis of iPSC conditioned medium via mass spectrometry. (Figure A) Series of various media used for the routine maintenance of iPS cells or for the induction of iPSC-conditioned medium were subjected to SDS-PAGE analysis. Coomassie brilliant blue staining revealed a robust band located between 55 and 70 kDa in iPSC-conditioned medium (lane 4). (Figure B) A sliced band from SDS-PAGE was in gel digested with trypsin following mass spectrometry. The Swiss-Prot database updated to March of 2015 was used to blast the resulting peptide fragments from the iPSC-conditioned medium.
(TIF)

S2 File iPSC-based treatments disturbed the phosphorylation of NF-κB. Cultured human PMNCs were polarized via LPS/IFNγ to proinflammatory M1 macrophages with robust phosphorylated NF-κB and recognized by the antiserum specific to the p65 subunit of NF-κB, which was phosphorylated at the Ser^536^ residue (Figure B). PMNCs without LPS/IFNγ stimulation served as negative controls of antiserum (Figure A). Compared with IKK2 inhibitor treatment (Figure C), which specifically suppressed the phosphorylation of NF-κB, either the iPSC CM (Figure D) or iPSCs (Figure E) treatments showed an inhibitory effect on NF-κB phosphorylation. The iPS cells were revealed via immunocytochemistry with anti-SSEA-1 antiserum (Figure E). Western blotting confirmed the immunocytochemistry result (Figure F).
(TIF)

S1 Table List of antibodies used. Detailed information of each antiserum used throughout the experiment.
(DOC)

S2 Table List of primer pairs used for semi-quantitative RT-PCR. Exclusive DNA sequences within the targeted gene were designated as a primer pair used in the semi-quantitative RT-PCR.
(DOC)

S3 Table Hemodynamic values of RV of MCT-induced PAH rats. The measurements of the RVSP and RV hypertrophy index of each group were represented as a table form. The data was showed of means ± SD. *p<0.05 vs. PBS group; ^#^*p<*0.05 vs. MCT group.
(TIF)
