# Supplementary figures and images for: Therapeutic Benefits of Induced Pluripotent Stem Cells in Monocrotaline-Induced Pulmonary Arterial Hypertension
Source: PLoS One. 2016 Feb 3;11(2):e0142476. doi: 10.1371/journal.pone.0142476 (PMC4740504; doi:10.1371/journal.pone.0142476)

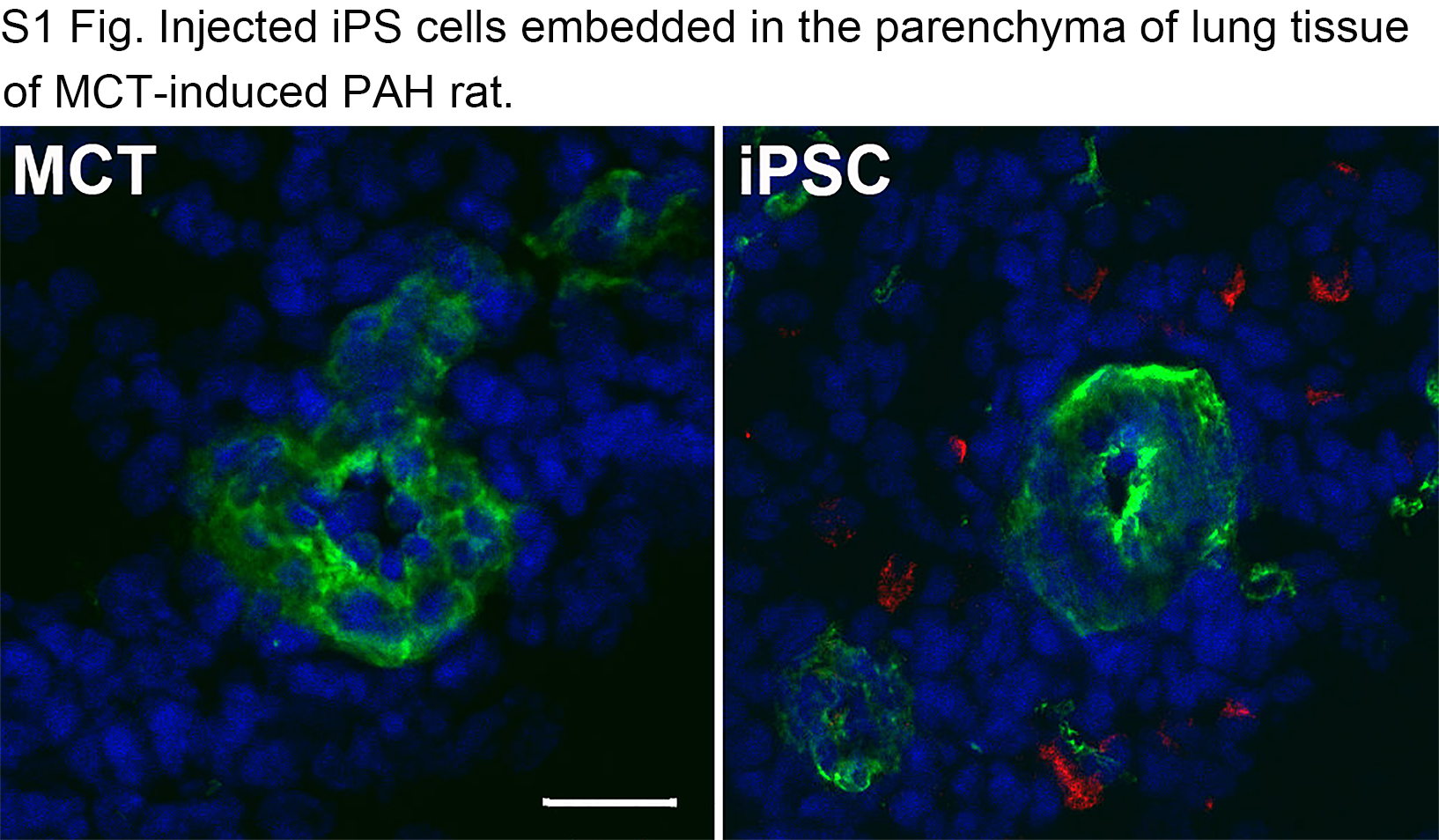

Supplement: S1 Fig — Four hours prior to sacrifice, iPS cells were injected into the MCT-induced PAH rat via tail vein. Lung tissue was harvested for frozen sectioning and immunohistochemistry. Specimens from the MCT-induced PAH rats without the injection of iPS cells served as an antiserum control. Thickening blood vessels due to hyperplasia of smooth muscle cells were revealed via intensive stain of α-smooth actin (FITC, left panel). In specimens from MCT-induced PAH rats receiving iPS cells injection, the iPS cells were recognized by the antisera against SOX2 protein (Texas Red, right panel). Scale bar = 20 μm. (TIF) [file pone.0142476.s001.tif]

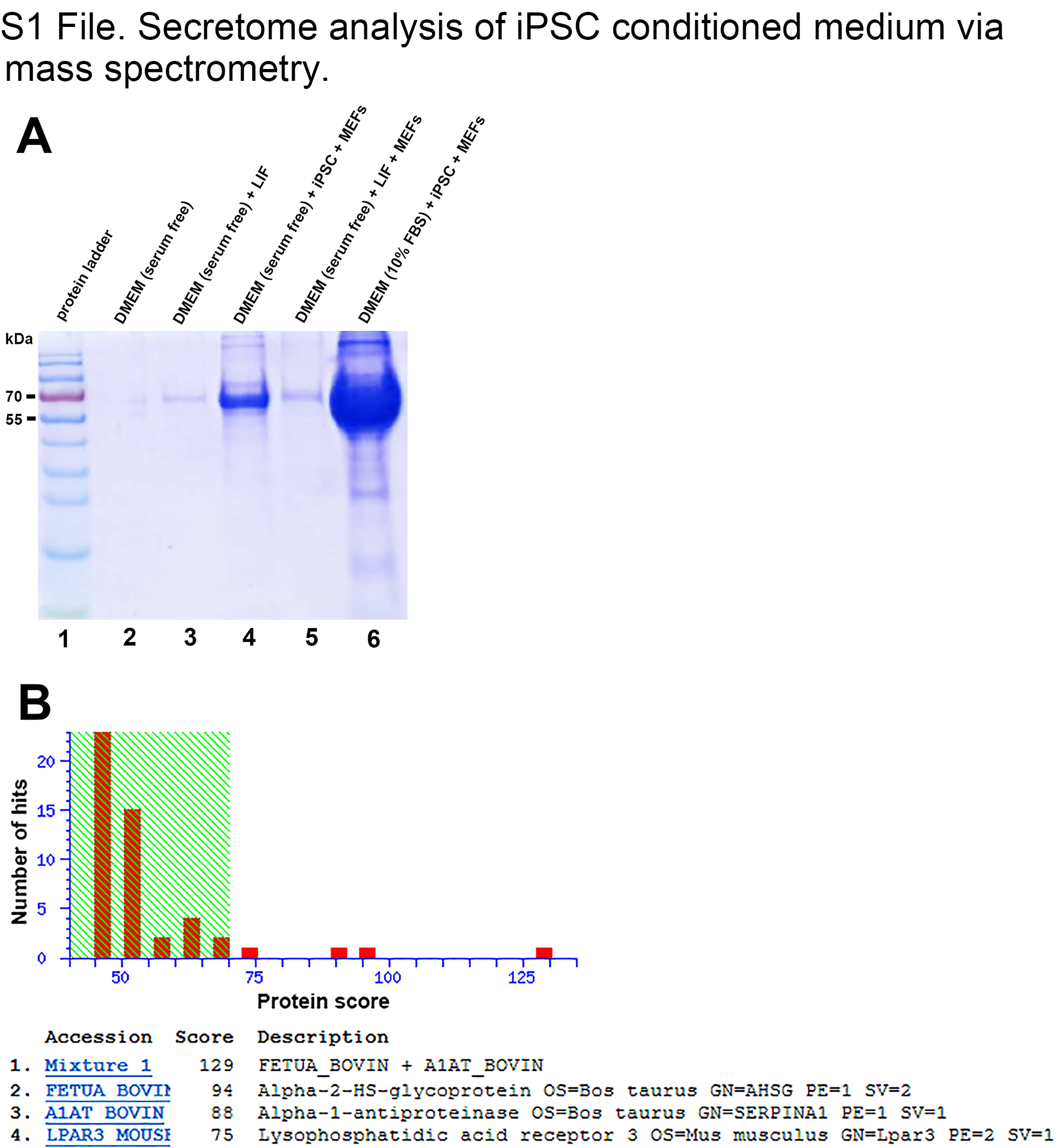

Supplement: S1 File — (Figure A) Series of various media used for the routine maintenance of iPS cells or for the induction of iPSC-conditioned medium were subjected to SDS-PAGE analysis. Coomassie brilliant blue staining revealed a robust band located between 55 and 70 kDa in iPSC-conditioned medium (lane 4). (Figure B) A sliced band from SDS-PAGE was in gel digested with trypsin following mass spectrometry. The Swiss-Prot database updated to March of 2015 was used to blast the resulting peptide fragments from the iPSC-conditioned medium. (TIF) [file pone.0142476.s002.tif]

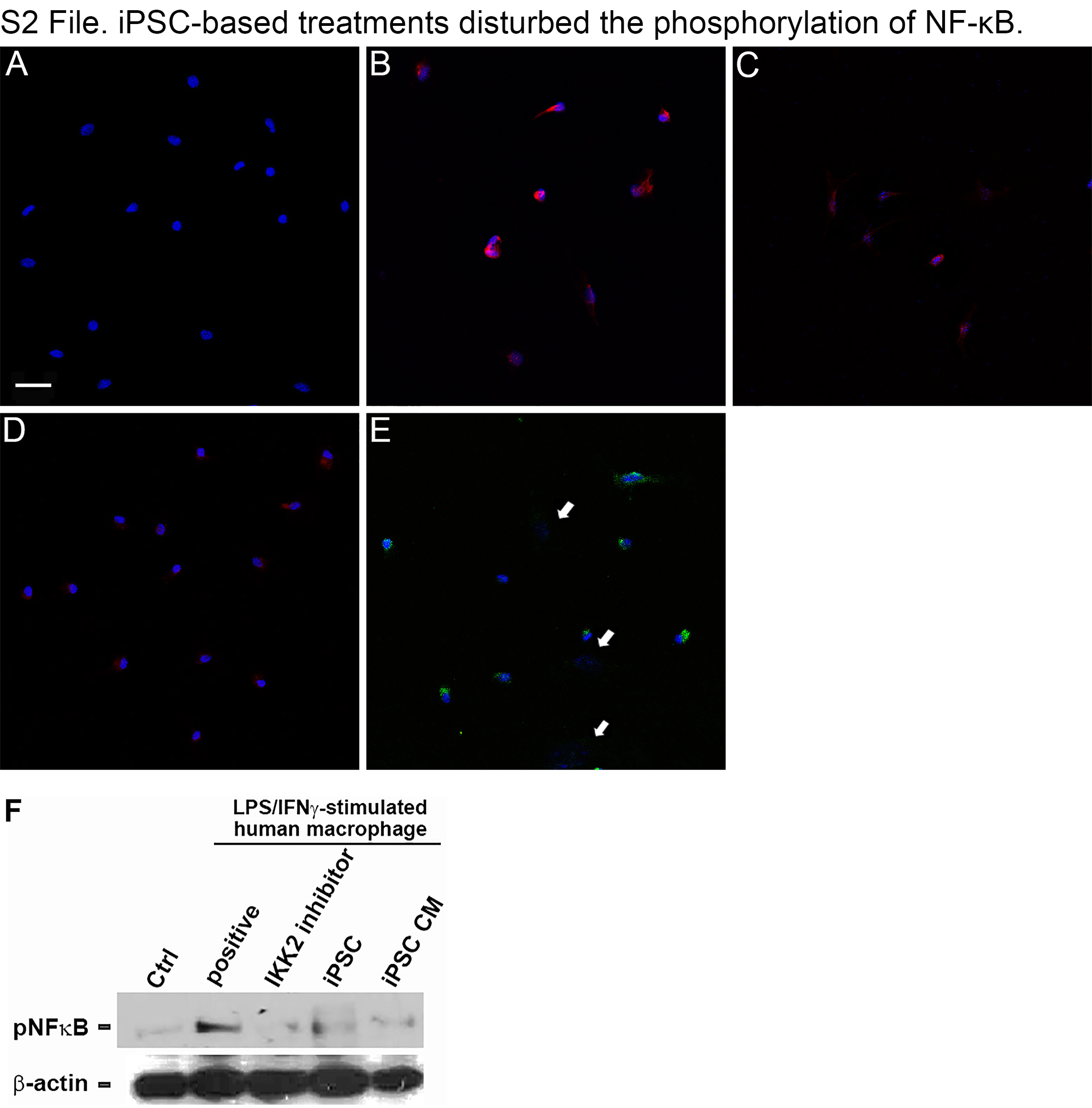

Supplement: S2 File — Cultured human PMNCs were polarized via LPS/IFNγ to proinflammatory M1 macrophages with robust phosphorylated NF-κB and recognized by the antiserum specific to the p65 subunit of NF-κB, which was phosphorylated at the Ser536 residue (Figure B). PMNCs without LPS/IFNγ stimulation served as negative controls of antiserum (Figure A). Compared with IKK2 inhibitor treatment (Figure C), which specifically suppressed the phosphorylation of NF-κB, either the iPSC CM (Figure D) or iPSCs (Figure E) treatments showed an inhibitory effect on NF-κB phosphorylation. The iPS cells were revealed via immunocytochemistry with anti-SSEA-1 antiserum (Figure E). Western blotting confirmed the immunocytochemistry result (Figure F). (TIF) [file pone.0142476.s003.tif]

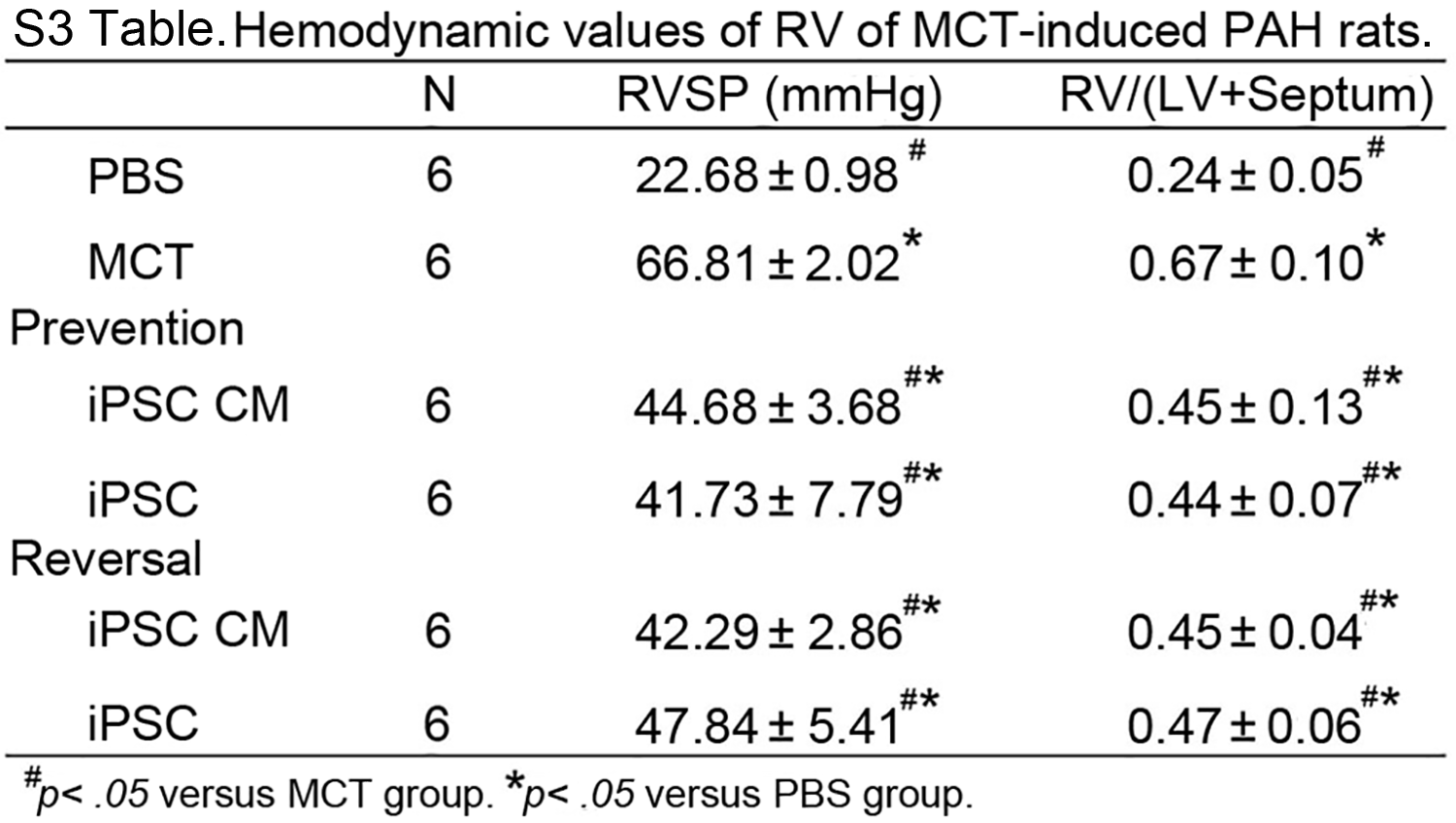

Supplement: S3 Table — The measurements of the RVSP and RV hypertrophy index of each group were represented as a table form. The data was showed of means ± SD. *p<0.05 vs. PBS group; #p<0.05 vs. MCT group. (TIF) [file pone.0142476.s006.tif]
