# Supplementary material for: Therapeutic Benefits of Induced Pluripotent Stem Cells in Monocrotaline-Induced Pulmonary Arterial Hypertension
Source: PLoS One. 2016 Feb 3;11(2):e0142476. doi: 10.1371/journal.pone.0142476 (PMC4740504; doi:10.1371/journal.pone.0142476)
Supplement: S1 Table — Detailed information of each antiserum used throughout the experiment. (DOC) [file pone.0142476.s004.doc]

**S1 Table. List of antibodies used in the article.**

| **Target** | **Type** | **Species** | **Catalog No.** | **Company** |
| --- | --- | --- | --- | --- |
| CD68 | monoclonal | mouse | ab31630 | Abcam |
| SSEA-1 | monoclonal | mouse | sc-21702 | Santa Cruz Biotechnology |
| NF-κB p65 | polyclonal | rabbit | sc-372 | Santa Cruz Biotechnology |
| phospho- NF-κB p65  (Ser536) | monoclonal | rabbit | #3033 | Cell Signaling Technology |
| MHC Class II | monoclonal | mouse | ab23990 | Abcam |
