# Supplementary material for: Therapeutic Benefits of Induced Pluripotent Stem Cells in Monocrotaline-Induced Pulmonary Arterial Hypertension
Source: PLoS One. 2016 Feb 3;11(2):e0142476. doi: 10.1371/journal.pone.0142476 (PMC4740504; doi:10.1371/journal.pone.0142476)
Supplement: S2 Table — Exclusive DNA sequences within the targeted gene were designated as a primer pair used in the semi-quantitative RT-PCR. (DOC) [file pone.0142476.s005.doc]

**S2 Table. List of primer pairs used for quantitative RT-PCR.**

| **Gene** |  | **Sequence (5' → 3')** |
| --- | --- | --- |
| *c-Myc* | Forward | CAACGTCTTGGAACGTCAGA |
|  | Reverse | CTCGTCTGCTTGAATGGACA |
| *Cripto* | Forward | ATGGACGCAACTGTGAACATGATGTTCGCA |
|  | Reverse | CTTTGAGGTCCTGGTCCATCACGTGACCAT |
| *Dax1* | Forward | TGCTGCGGTCCAGGCCATCAAGAG |
|  | Reverse | GGGCACTGTTCAGTTCAGCGGATC |
| *Eras* | Forward | ACTGCCCCTCATCAGACTGCTACT |
|  | Reverse | CACTGCCTTGTACTCGGGTAGCTG |
| *Fbx15* | Forward | GGTGGGGCTGTGGCAGGAGA |
|  | Reverse | AGAGTAAGCCGGCTGCGGGA |
| *Fgf4* | Forward | CGTGGTGAGCATCTTCGGAGTGG |
|  | Reverse | CCTTCTTGGTCCGCCCGTTCTTA |
| *IL-1* | Forward | TCCTCTGTGACTCGTGGGAT |
|  | Reverse | TCAGACAGCACGAGGCATTT |
| *IL-6* | Forward | CACTTCACAAGTCGGAGGCT |
|  | Reverse | TCTGACAGTGCATCATCGCT |
| *IL-12α   * | Forward | CCGGTCCAGCATGTGTCAAT |
|  | Reverse | CACTTGGCAGGTCCAGAGAC |
| *IL-12β   * | Forward | ATCATCAAACCGGACCCACC |
|  | Reverse | CAGGAGTCAGGGTACTCCCA |
| *IL-23* | Forward | CATACCTCCCTACTGGGCCT |
|  | Reverse | GAGAAGAGAACGCTGCCACT |
| *INFγ  * | Forward | CCCTCTCTGGCTGTTACTGC |
|  | Reverse | TTTCGTGTTACCGTCCTTTTG |
| *Klf4* | Forward | GCAGTCACAAGTCCCCTCTC |
|  | Reverse | CTGTGTGAGTTCGCAGGTGT |
| *Nanog* | Forward | GGAACGCCTCATCAATGC |
|  | Reverse | TTTGTTTGGGACTGGTAGAAG |
| *Nat1* | Forward | ATTCTTCGTTGTCAAGCCGCCAAAGTGGAG |
|  | Reverse | AGTTGTTTGCTGCGGAGTTGTCATCTCGTC |
| *NF-κB p65* | Forward | TCTGCTTCCAGGTGACAGTG |
|  | Reverse | ATCTTGAGCTCGGCAGTGTT |
| *Oct4* | Forward | GCATACGAGTTCTGCGGAGG |
|  | Reverse | TCTCCAACTTCACGGCATTG |
| *Rex1* | Forward | GCCAGCAGCTCCTGCACACA |
|  | Reverse | TGAGCTCGCCCCAACCCTCA |
| *Sox2* | Forward | CAGGAGAACCCCAAGATG |
|  | Reverse | GTGTACTTATCCTTCTTC |
| *Zfp296* | Forward | CCATTAGGGGCCATCATCGCTTTC |
|  | Reverse | CACTGCTCACTGGAGGGGGCTTGC |
| *36b4* | Forward | GCCAGCGAAGCCACGCTGCTGAAC |
|  | Reverse | CGAACACCTGCTGGATGACCAGCCC |
